# Rapid Effect of Nicotine Intake on Neuroplasticity in Non-Smoking Humans

**DOI:** 10.3389/fphar.2012.00186

**Published:** 2012-10-26

**Authors:** Jessica Grundey, Nivethida Thirugnanasambandam, Kim Kaminsky, Anne Drees, Angela C. Skwirba, Nicolas Lang, Walter Paulus, Michael A. Nitsche

**Affiliations:** ^1^Department of Clinical Neurophysiology, Georg-August-UniversityGöttingen, Germany; ^2^Department of Neurology, Christian-Albrechts UniversityKiel, Germany

**Keywords:** neuroplasticity, nicotine, non-smokers, PAS, tDCS

## Abstract

In various studies nicotine has shown to alter cognitive functions in non-smoking subjects. The physiological basis for these effects might be nicotine-generated modulation of cortical structure, excitability, and activity, as mainly described in animal experiments. In accordance, a recently conducted study demonstrated that application of nicotine for hours via nicotine patch in non-smoking humans alters the effects of neuroplasticity-inducing non-invasive brain stimulation techniques on cortical excitability. Specifically, nicotine abolished inhibitory plasticity independent from the focality of the stimulation protocol. While nicotine prevented also the establishment of non-focal facilitatory plasticity, focal synapse-specific facilitatory plasticity was enhanced. These results agree with a focusing effect of prolonged nicotine application on facilitatory plasticity. However, since nicotine induces rapid adaption processes of its receptors, this scenario might differ from the effect of nicotine in cigarette smoking. Thus in this study we aimed to gain further insight in the mechanism of nicotine on plasticity by exploring the effect of nicotine spray on non-focal and focal plasticity-inducing protocols in non-smoking subjects, a fast-acting agent better comparable to cigarette smoking. Focal, synapse-specific plasticity was induced by paired associative stimulation (PAS), while non-focal plasticity was elicited by transcranial direct current stimulation (tDCS). Forty eight non-smokers received nicotine spray respectively placebo combined with one of the following protocols (anodal tDCS, cathodal tDCS, PAS-25, and PAS-10). Corticospinal excitability was monitored via motor-evoked potentials elicited by transcranial magnetic stimulation (TMS). Nicotine spray abolished facilitatory plasticity irrespective of focality and PAS-10-induced excitability diminution, while tDCS-derived excitability reduction was delayed and weakened. Nicotine spray had thus a clear effect on neuroplasticity in non-smoking subjects. However, the effects of nicotine spray differ clearly from those of prolonged nicotine application, which might be due to missing adaptive nicotinic receptor alterations. These results enhance our knowledge about the dynamic impact of nicotine on plasticity, which might be related to its heterogenous effect on cognition.

## Introduction

Nicotine binds to the nicotinergic type of cholinergic receptors, which are ligand-gated cation channels. Nicotine also affects other transmitter systems by regulating the release of dopamine, adrenaline, serotonin, and glutamate, amongst others. Hereby nicotine is thought to be critically involved in the induction and modulation of neuroplasticity (Burnashev, 1998; Dajas-Bailador and Wonnacott, 2004; Levin et al., 2006), the likely physiological basis of learning and memory formation (Rioult-Pedotti et al., 1998, 2000). Specifically, in animal experiments the activation of nicotinic receptors results in a facilitation of long-term potentation (LTP), both dependent and independent of NMDA-receptor activation (Sawada et al., 1994; Huerta and Lisman, 1995; Matsuyama et al., 2000).

### Cognitive effects of nicotine

On a functional basis, in animal experiments nicotine has been shown to improve working memory function (Levin et al., 1994) and attention (Hahn and Stolerman, 2002), while in humans cognitive results are heterogeneous. Kleykamp et al. (2005) have found no effect of nicotine gum in different doses on attention and working memory in never-smokers. In contrast, other studies have shown that nicotine improves alerting attention-accuracy (Barr et al., 2008), visuospatial attention (Thiel et al., 2005), and working memory (Kumari et al., 2003) in non-smoking subjects. However, nicotinic plasticity modulation has been explored only in few studies in humans so far.

### Nicotinic plasticity modulation

Recently it was shown that global cholinergic activation via the cholinesterase-inhibitor rivastigmine enhances focal plasticity induced by paired associative stimulation (PAS) and abolishes/reverses non-focal facilitatory plasticity generated by transcranial direct current stimulation (tDCS), thus resulting in a focusing effect of acetylcholine on facilitatory plasticity (Kuo et al., 2007). Similar results have been found for nicotinic effects in non-smokers (Thirugnanasambandam et al., 2011). However, in difference to global cholinergic activation, nicotine reduced, or abolished inhibitory plasticity in these subjects. In principal accordance, nicotine enhanced the facilitatory effects of intermittent theta burst stimulation in the human motor cortex (Swayne et al., 2009). However, nicotine pharmacokinetics in the above-mentioned study of Thirugnanasambandam et al. (2011) differ from that present in cigarette smokers, because in that study nicotine patches were applied, which are characterized by a slow build-up and long duration of enhanced nicotine concentration, whereas cigarette smoking results in a fast build-up and decay of nicotine concentration. Since nicotinic receptor activation induces rapid adaptive processes like desensitization (Alkondon et al., 2000; Mansvelder and McGehee, 2000), upregulation, and greater density of AChR (Flores et al., 1992; Mukhin et al., 2008) these might have affected the impact of nicotine on plasticity relevantly in that study.

### Plasticity induction protocols

In the present study, we therefore aimed to mimic the pharmacokinetics of cigarette smoking by exploring the impact of nicotine spray, which results in maximal nicotine plasma concentration within a few minutes, on focal and non-focal plasticity. Facilitatory and inhibitory plasticity were induced by tDCS (Nitsche and Paulus, 2000, 2001) and PAS (Stefan et al., 2000). Both stimulation protocols induce non-invasively NMDA- and calcium channel-dependent plasticity (Stefan et al., 2002; Nitsche et al., 2003, 2004), though tDCS is supposed to induce non-focal plasticity changes due to affecting large neuron populations under large electrodes (Purpura and McMurtry, 1965; Nitsche et al., 2007), while PAS-induced plasticity is restricted to synaptic connections between somatosensory and motor cortex (Weise et al., 2006). As outlined above, in non-smokers nicotine patch abolished inhibitory plasticity and focused facilitatory plasticity. Since nicotine receptors are rapidly modified by chronic nicotine exposure, we hypothesize that administration of a single dose of fast-acting nicotine might affect plasticity differently as compared to nicotine patch.

## Materials and Methods

### Subjects

Altogether 48 otherwise healthy non-smoking subjects participated in this study. Table 1 displays the characteristics of the subjects in terms of age and gender. All subjects were of Caucasian origin. Chronic and acute medical diseases or any history of neurological/psychiatric disease were excluded before entering the study by assessment of medical history, likewise intake of chronic and acute medication. Pregnancy, family history of epilepsy, presence of any metallic implant, or cardiac pacemaker were ruled out. All subjects gave written informed consent before participating in the study. The experiments were approved by the local Ethics Committee and conformed to the principles laid down in the Declaration of Helsinki. Allocation of the subjects to the respective experimental conditions as well as order of sessions was randomized.

**Table 1:** **Characteristics of the subjects participating in the experiments**.

Stimulation Parameter	Anodal tDCS	Cathodal tDCS	PAS-10	PAS-25
Number of subjects	12	12	12	12
Number of females(%)	6(50)	7(58)	6(50)	6(50)
Age	24.4 ± 1.2	26.9 ± 3.6	25.9 ± 2.1	24.5 ± 1.3
S1 mV before nicotine spray	41.3	45.6	43.9	43.6
S1 mV after nicotine spray	42.3	44.9	44.4	43.3

### Paired associative stimulation

Altogether twenty-four subjects participated in the PAS experiment. Twelve non-smokers participated in the inhibitory PAS protocol (PAS-10) and 12 in the excitatory PAS protocol (PAS-25). Peripheral nerve stimulation was delivered to the right ulnar nerve at the wrist level by a Digitimer D185 multipulse stimulator (Digitimer, Welwyn Garden City, UK) at an intensity of 300% of the sensory perceptual threshold followed by single pulse transcranial magnetic stimulation (TMS) applied with a stimulator output resulting in motor-evoked potentials (MEPs) of approximately 1 mV amplitude (“baseline intensity,” see description in Section “Monitoring of Cortical Excitability”). The paired pulses were repeated 90 times at a frequency of 0.05 Hz. This protocol induces long-lasting excitability changes in the motor cortex depending on the interstimulus interval (ISI). An ISI of 10 ms induces excitability diminution (PAS-10) whereas an ISI of 25 ms induces facilitation (PAS-25; Stefan et al., 2000; Wolters et al., 2003). The PAS-protocols were combined with either nicotine or placebo spray for each subject in different experimental sessions.

### Transcranial direct current stimulation

Twenty four subjects participated in the tDCS experiments (12 non-smoking subjects participated in the inhibitory tDCS protocol and 12 in the excitatory tDCS protocol). We used a battery-driven constant current stimulator (Schneider Electronics, Gleichen, Germany) with a maximum output of 2 mA. tDCS was administered via rubber electrodes covered by saline soaked sponges (35 cm^2^). One electrode was positioned over the motor cortex representational area of the right abductor digiti minimi muscle (ADM), the other electrode above the right orbit. All subjects received 1 mA of either anodal or cathodal stimulation for 13 min (anodal tDCS) or 9 min (cathodal tDCS), which had been demonstrated to induce cortical excitability enhancement or inhibition lasting for about 1 h after the end of stimulation (Nitsche and Paulus, 2001; Nitsche et al., 2003) combined with nicotine spray or placebo medication in different experimental sessions.

#### Monitoring of motor cortex excitability

Transcranial magnetic stimulation-elicited MEPs were recorded to measure excitability changes of the representational motor cortical area of the right ADM. Single pulse TMS was conducted by a Magstim 200 magnetic stimulator (Magstim Company, Whitland, Dyfed, UK) at a frequency of 0.25 Hz with a figure of eight-shaped coil (diameter of one winding 70 mm, peak magnetic field, 2.2 T). The coil was held tangentially to the scalp at an angle of 45° to the sagittal plane with the coil handle pointing laterally and posterior. The optimal position was defined as the site where stimulation resulted consistently in the largest MEPs. Surface EMG was recorded from the right ADM with Ag–AgCl electrodes in a belly tendon montage. The signals were amplified and filtered with a time constant of 10 ms and a low-pass filter of 2.5 kHz, then digitized at an analog-to-digital rate of 5 kHz and further relayed into a laboratory computer using the Signal software and CED 1401 hardware (Cambridge Electronic Design). The intensity was adjusted to elicit, on average, baseline MEPs of 1 mV peak-to-peak amplitude, and was kept constant for the post-stimulation.

### Pharmacological intervention

Each subject participated in two sessions in randomized order. Nasal spray contained nicotine or inactive placebo. Nicotine was administered by nasal spray, containing 10 mg/ml nicotine, in a cumulative dose of 1 mg (Nicorette ^®^Nasal Spray, McNeil Products, UK) to all subjects in combination with one of the stimulation protocols, anodal tDCS, cathodal tDCS, PAS-10, and PAS-25. The rise time of nicotine by nasal spray in venous blood levels is close to venous blood levels of nicotine delivered by cigarets (Schneider et al., 1995) with a plasma peak level after 5–10 min. Side effects of the nasal nicotinic administration were coughing, sneezing, throat irritation, and dizziness like it has been described from prior clinical trials (Sutherland et al., 1992). Symptoms subsided rapidly after some minutes. To have a comparability to the nicotine patch study, we chose a dose of nicotine spray (1 mg) that delivers nicotine blood levels comparable to those of nicotine patch (8–9 ng/ml, see also Tønnesen et al., 1991; Pomerleau et al., 1992).

### Course of the experiment

**Figure 1:**
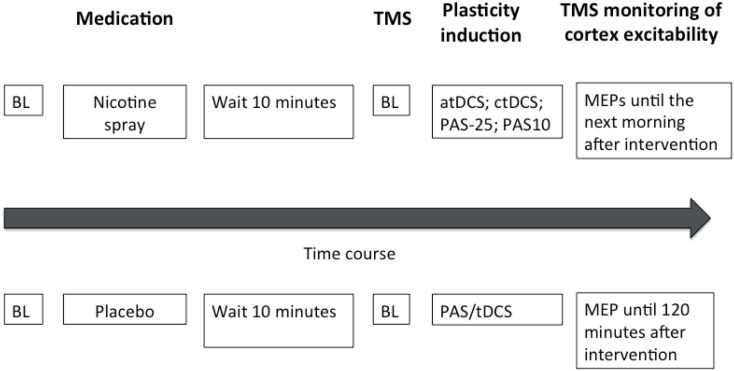
**This figure illustrates the experimental protocol of our study**. At the beginning of the session baseline measurements (BL) were performed and followed by administration of either nicotine spray or placebo spray. After 10 min baseline (BL) motor cortical excitability was redetermined via TMS-induced motor-evoked potentials (MEP). One of the four excitability-inducing protocols were then applied (atDCS, ctDCS, PAS-10, PAS-25). After-measurements started immediately after the application of the protocols, and were conducted every 5 min for the first 30 min, then every 30 min up to 120 min for both sessions. MEP amplitudes were also recorded the next morning and next evening for the nicotine spray sessions.

Subjects were seated comfortably in a reclined chair with head- and armrest and asked to relax completely. EMG electrodes were placed at the right ADM as described above. Their exact position was marked with a pen. Then TMS was applied over the left representational area of the right ADM to determine the spot with the consistently highest MEPs in the resting ADM (optimal site), which was then marked with a pen. The TMS-intensity was adjusted to elicit MEP amplitudes of 1 mV (S1 mV). Twenty MEPs were recorded at this stimulus intensity and the mean MEP amplitude was calculated at baseline. Then nicotine nasal spray respective placebo spray was administered. Side effects like coughing and sneezing, that could interfere with the measurements subsided quickly and 10 min later one of the stimulation protocols, either tDCS or PAS, was administered, followed by immediate recording of at least 20 MEPs at the time points of 0, 5, 10, 15, 20, 25, 30, 60, 90, and 120 min. For the nicotine spray condition, the after-measurements were also conducted the evening of the stimulation day and in the morning and evening of the day following the plasticity induction procedure. Sessions were conducted in randomized order, and an inter-session interval of at least 1 week was obligatory to avoid interferences. See also Figure 1 for course of the experiment.

### Data analysis and statistics

For all subjects the means of 20 MEP amplitudes recorded at each time point was calculated. The post-intervention mean MEP amplitudes from each subject were then normalized to the respective individual mean baseline MEP amplitude (quotient of post- vs. pre-intervention MEP amplitudes). Statistical analysis used SPSS general linear model analysis for variances for repeated measurements on normalized data. MEP amplitude was the dependent variable including all time points up to 120 min after stimulation. Drug (nicotine vs. plc) and time points were included as within-subjects factors. Stimulation protocol (atDCS, ctDCS, PAS-25, and PAS-10) served as between-subject factors. Mauchly’s sphericity test was performed and Greenhouse–Geisser correction was applied when necessary. Student’s *t*-tests (paired samples, two-tailed, *p* < 0.05, not adjusted for multiple comparisons) were performed to compare the MEP amplitudes before and after the interventional brain stimulations in each condition and between drug conditions (nicotine/placebo) for each time point. A *p*-value of <0.05 was considered significant for all statistical analyses. Significances of differences in demographic factors were tested by one-way ANOVA and chi-square for gender. All data are expressed as mean ± standard of error (SEM).

## Results

All subjects tolerated the experiments well, even though nearly all of the subjects (44 of 46 subjects, 96%) complaint about sneezing and coughing after inhalation of nicotine spray. No significant group differences were found in terms of age, gender, TMS-intensity to elicit an MEP of 1 mV (S1 mV) before and after administration of nicotine spray (see Table 1). Absolute baseline MEP amplitudes did not differ significantly within and between stimulation groups and medication conditions (Student’s *t*-test, two-tailed, unpaired/paired, *p* > 0.05 for all cases). The ANOVA revealed a significant main effect of the between-subject factor timepoint and stimulation. The interactions drug × stimulation, timepoint × stimulation, and drug × timepoint × stimulation were also significant (see also Table 2 for results of degrees of freedom, *F*-value, *p*-value, *indicates significant values with *p* < 0.05).

**Table 2:** **Results of the ANOVA**.

Parameters	*df*	*F*-Value	*p*-value
Drug	1	2.288	0.138
Timepoint	6.932	2.163	0.038*
Stimulation	3	11.423	0.001*
Drug × stimulation	3	8.673	0.001*
Timepoint × stimulation	30	2.056	0.001*
Drug × timepoint	5.446	1.478	0.192
Drug × timepoint × stimulation	30	1.831	0.027*

*p < 0.05

### Effects of nicotine spray on tDCS-induced plasticity in non-smokers

**Figure 2:**
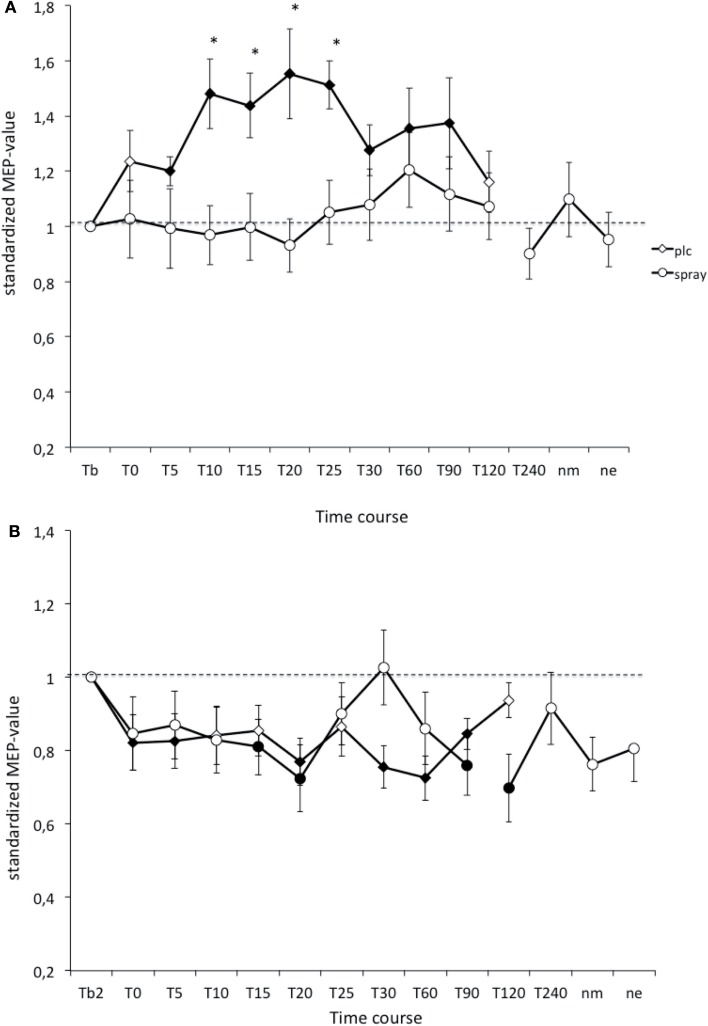
**(A,B)** Nicotinergic impact on transcranial direct current stimulation (tDCS) induced neuroplasticity. Shown are the graphs with motor evoked potentials (MEP) standardized to the baseline on the *Y*-axis plotted against different time points post-intervention on the *X*-axis. Filled symbols indicate statistically significant deviations from baseline and asterisks indicate significant differences between the control and nicotine conditions (Student’s *t*-test, paired, two-tailed, *p* < 0.05). nm, Next morning; ne, next evening; plc, placebo. Tb, Baseline MEP-amplitude before begin of the stimulation protocols (standardized). Error bars indicate standard error of mean.

In the PLC condition the anodal tDCS-induced excitability increased MEP amplitudes stayed significant until 90 min after stimulation, and the cathodal tDCS-induced inhibition lasted until 90 min after tDCS. As revealed by the *post hoc* *t*-test (paired, two-tailed, *p* < 0.05) nicotine spray abolished the atDCS-induced long-lasting excitability enhancements in non-smoking subjects. For the cathodal tDCS protocol under influence of nicotine spray excitability diminuation started delayed after 15 min and lasted only until 20 min after stimulation. A second diminuation peak could be seen after 90 min and lasted until 120 min post-stimulation (Figure 2). Thus nicotine spray administration delayed and weakened the ctDCS-induced after-effects.

### Effects of nicotine spray on PAS-induced plasticity in non-smokers

**Figure 3:**
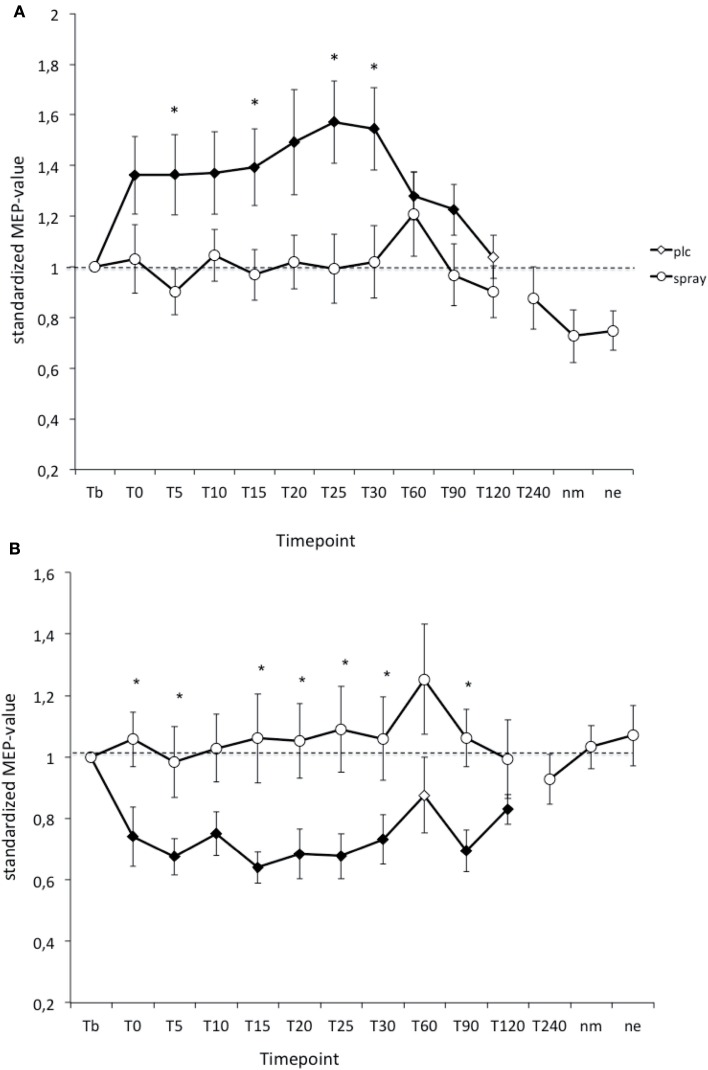
**(A,B)** Nicotinergic impact on paired associative stimulation (PAS) induced neuroplasticity. Shown are the graphs with motor evoked potentials (MEP) standardized to the baseline on the *Y*-axis plotted against different time points post-stimulation on the *X*-axis. Filled symbols indicate statistically significant deviations from baseline and asterisks indicate significant differences between the placebo medication and nicotine conditions (Student’s *t*-test, paired, two-tailed, *p* < 0.05). nm, Next morning; ne, next evening; plc, placebo. Tb, Baseline MEP-amplitude before begin of the stimulation protocols (standardized). Error bars indicate standard error of mean.

As revealed by Student’s *t*-test (paired, two-tailed, *p* < 0.05) in the PLC- condition PAS-25-induced excitability changes (MEP-enhancements) were significantly increased for up to 90 min after stimulation, while the PAS-10-induced long-lasting after-effects returned to baseline 120 min after PAS. Nicotine spray abolished both the PAS-10-induced inhibitory MEP-changes and the PAS-25-induced excitatory after-effects completely (Figure 3). Thus nicotine spray prevented the induction of PAS-induced focal plasticity.

### Effect of nicotine spray on motor cortex excitability measured by TMS-elicited motor-evoked potentials

**Figure 4:**
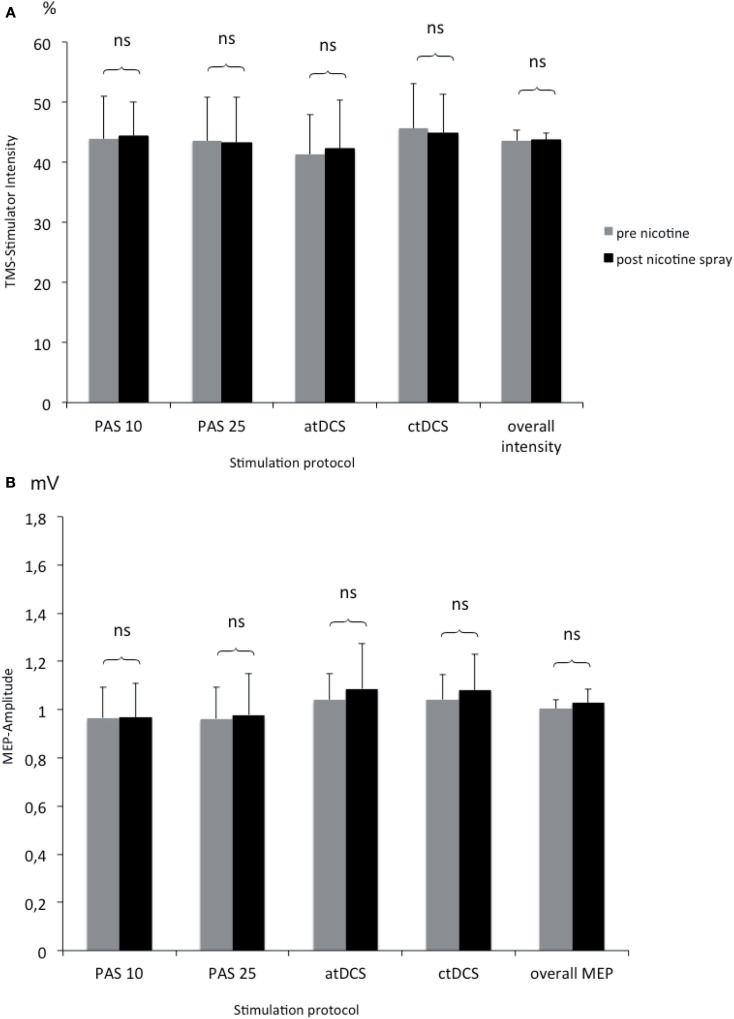
**(A,B)** Comparison of TMS-stimulator intensity and MEP amplitudes before and after nicotine spray administration. Shown are the TMS-stimulator intensity as percentage of maximum stimulator output **(A)** and respective motor evoked potentials [MEP amplitudes in mV; **(B)**] before and after nicotine spray administration for the different interventions (atDCS, ctDCS, PAS-25, PAS-10). ns, Non significant; mV, millivolt.

To rule out, that nicotine spray itself increases or decreases motor cortex excitability measured by TMS, we compared TMS-intensity needed to elicit an MEP of 1 mV before and after the administration of nicotine spray. The respective Student’s *t*-test (paired, two-tailed) did not reveal any significant difference between different stimulation groups and before vs. after nicotine (see Figure 4).

## Discussion

The results of the present study show that nicotine administration has a prominent and rapidly evolving effect on stimulation-induced neuroplasticity in the human primary motor cortex. In non-smoking humans, nicotine spray prevented completely the induction of focal and non-focal plasticity, as induced by PAS-25, and anodal tDCS. Moreover it reduced excitability-diminishing plasticity accomplished by PAS-10, whereas nicotine had no major impact on cathodal tDCS-generated plasticity.

These results are only in partial accordance with those of a former study in our group, where we explored the effects of long-acting nicotine, administered via patch, on plasticity induced by the same stimulation techniques (Thirugnanasambandam et al., 2011). Here nicotine abolished inhibitory plasticity regardless of its focality, whereas facilitatory plasticity was enhanced, when the induction procedure was focal, but abolished, when it was non-focal. Moreover, the abolishing effect of nicotine spray on facilitatory plasticity in the present study differs prominently from the effects of nicotine lozenge application, which enhanced facilitatory plasticity induced by intermittent theta burst stimulation of the human motor cortex (Swayne et al., 2009). The comparison between the present study and that of Thirugnanasambandam, in which resulting nicotine plasma level should be more or less identical, is in favor for an impact of the duration of nicotine application on human cortical plasticity. One reason for these different effects of acute (in terms of minutes), and chronic (in terms of hours) nicotine administration on plasticity might be adaptive receptor up- or downregulation, which takes place rapidly in nicotinic receptors (Flores et al., 1992; Alkondon et al., 2000; Mukhin et al., 2008; Mansvelder and McGehee 2000) and thus might have had an impact on the results of the patch study. With regard to the study of Swayne and co-workers, some other aspects differ between the respective studies, such as kind of stimulation protocol, duration of plasticity induced by the stimulation protocol alone, expected plasma level, and pharmacokinetics, which makes a comparison between studies difficult, but nevertheless is in advance for a neuromodulatory effect of nicotine on plasticity, whose direction might be determined by diverse factors.

### Proposed mechanism of action

The results of the present study allow no definite conclusions about how nicotine has affected plasticity in the present study, but some candidate mechanisms can be derived from the more general functions of nicotinic receptors, and the physiological basis of tDCS, and PAS. Nicotine binds to nicotinergic ACh-receptors that are widely distributed throughout the brain (Albuquerque et al., 2009). In the brain two major subunits compositions exist, the heteromeric assembly of α4β2 and the homomeric α7 subunit, both exhibiting different pharmacological and physiological properties (Jones et al., 1999), and both increasing intracellular calcium levels by serving as pre- and postsynaptic ligand-gated calcium channels. As the after-effects of tDCS and PAS are likewise calcium-dependent (Stefan et al., 2002; Nitsche et al., 2003), a possible effect of nicotine on neuroplasticity might be alteration of intracellular calcium levels. The amount of intracellular calcium determines if inhibitory, facilitatory, or no plasticity is induced (Lisman, 2001; Misonou et al., 2004). Given that nicotine enhances intracellular calcium concentration via activation of the respective nicotinic receptors, nicotine administration might have caused a calcium concentration that overshoots the limit for LTP-like induction elicited by atDCS and PAS-25, thus resulting in an extinction of after-effects. The same might be true for LTD-induction processes.

This proposed mechanism of action is however hypothetical and highly speculative presently and needs to be further explored experimentally in the future. Due to the complex impact of nicotine on other neuromodulators and – transmitters, alternative mechanisms of action cannot be ruled out.

### General remarks

The results of our study demonstrate that nicotine spray influences neuroplasticity in non-smoking humans prominently. Nicotine spray in abolishes/reduces focal and non-focal plasticity. Here the connection to cognitive studies is not easily drawn, since the nicotinic effect on cognition and memory in non-smokers is discussed controversially. A review of Heishman (1998) reported about no true enhancement of sensory ability, selective attention, learning, and other cognitive abilities (e.g., problem solving, reasoning) in non-smoking subjects. Ernst et al. (2001) describe a nicotinic improvement of reaction time, but no effect on working memory in non-smokers. Poltavski and Petros (2005) have even shown a decrement in working memory in non-smokers. To further complicate argumentation the majority of the studies have used nicotine devices other than nicotine spray (nicotine patch, cigarets, inhaler), thus comparability to these studies is difficult because of different bioavailability. Only few studies have used nicotine spray in non-smokers and have found amelioration in rapid visual information processing (Rusted and Alvares, 2008) and fine motor-skills (Perkins et al., 2008). Potential connections of nicotinergic changes in LTP- and LTP-like plasticity and alterations in cognitive functions in smokers and non-smokers have to be explored more directly and intensively in the future. To clarify the specific receptor mechanisms, different nicotinic receptor subtypes have to be examined by pharmacological interventions (agonists and antagonists). Moreover further exploration of genetic differences between smokers and non-smokers might give further insight into the question, why people develop a nicotine addiction.

### Limiting conditions

Some limitations of the present study should be taken into account. The study was conducted in a single-blinded manner, so that the person carrying out the experiments knew about the condition of the subject (nicotine spray vs. placebo), thus delivering a possible confounding factor. Furthermore, TMS-measurements of cortical excitability were not performed until the next day in the placebo group, which complicates evaluation of nicotinic long-lasting effects on plasticity. Another limitation is the fact, that we cannot exclude non-linear dose-dependent effects of nicotine spray, since we performed the study with a stable dose of 1 mg. Dose-related effects on cortical excitability have p.e. been described for dopamine (Monte-Silva et al., 2009) and possible have to be taken into account also for other substances. This might be of special importance for the physiological effects of neuromodulators. Another restriction is that we did not obtain blood levels of nicotine, thus it cannot be excluded completely that different effects of nicotine patch and spray applications on plasticity are at least partially caused by different blood concentrations. It might furthermore complicate the comparability of the results of this study with other studies as well as cognitive testing. Still former pharmacological studies have measured blood levels in subjects taking 1 mg nicotine spray. With an average of 8–9 ng/ml, the blood level did not differ from the blood levels obtained after administration of nicotine patch 15 mg/16 h (8,9 ng/ml), which is the dosage we had chosen for the patch study (Tønnesen et al., 1991; Pomerleau et al., 1992; Thirugnanasambandam et al., 2011).

Moreover it should be mentioned that the explanation and discussion of the results is only hypothetical presently as currently direct correlation to cognitive studies is still missing. Possible future studies might test this linking between cortical excitability changes and impact on cognitive functions more directly and give further insight in the mechanisms of nicotine in smokers and non-smokers. Since chronic nicotine spray administration has fast and similar effects on craving and withdrawal symptoms than patch (Hajek et al., 1999) it would be further interesting in future possible studies to explore long-term effects of nicotine spray on neuroplasticity.

## Conflict of Interest Statement

The authors declare that the research was conducted in the absence of any commercial or financial relationships that could be construed as a potential conflict of interest.
